# Can UPR integrate fasting and stem cell regeneration?

**DOI:** 10.3389/fchem.2015.00005

**Published:** 2015-02-03

**Authors:** Ruchi Chaube

**Affiliations:** Department of Medicine and Institute for Transformative Molecular Medicine, Case Western Reserve University School of Medicine and University HospitalsCleveland, OH, USA

**Keywords:** fasting, upr, er stress, stem cell regeneration, PKA, IGF1 signaling

In order to find ways to counter massive loss of regeneration and self-renewal that the stem cells (SCs) suffer from following chemotherapy, the cancer biologists have set foot in regenerative medicine. A significant breakthrough in this direction, made by Dr. Longo's group situated at University of Southern California, has demonstrated that cycles of prolonged fasting (72 h and more) and re-feeding prior to chemotherapy protected hematopoietic stem cells (HSCs) from damage and promoted their self-renewal and regeneration: a potential benefit following chemotherapy (Cheng et al., 2014). While prolonged fasting may not be an attractive and practical option for the debilitated and the elderly, further research into the phenomenon can lead to the discovery of molecular targets which can mimic the similar effect without food deprivation. In the Longo's study, the pro-regenerative effects of fasting were shown to be mediated by a reduction in PKA activity and IGF1 levels within the HSCs microenvironment. However, PKA and IGF1 interact with a myriad of signaling pathways, and the concrete targets, which prevent the loss of regeneration in SCs from prolonged fasting, are yet to be identified.

An alternate view of this observation revolves around this question: what is the state of cellular stress under fasting? During glucose starvation, a condition analogous to fasting, the cells undergo stress, and cellular stresses collectively give rise to unfolded protein response (UPR) in the endoplasmic reticulum (ER) and, subsequently, ER stress. Moreover, cancer cells voraciously rely on glucose from the bloodstream for energy generation and proliferation, and glucose deprived cancer cells undergo death by UPR mediated mechanisms (Palorini et al., 2013). The role of the IRE1 signaling branch of UPR and its downstream target Xbp1 is of note here because it integrates both cancer and fasting (glucose starvation). IRE1 is the most conserved branch of UPR signaling from the yeast to the metazoans; autophosphorylation activates it and triggers its endoribonuclease activity on its primary target, the mRNA of X-box binding protein1 (Xbp1). The spliced Xbp1, referred to as Xbp1s, translates into a transcription factor and stimulates genes for the chaperones and components of the ER-associated protein degradation pathway. It has been shown recently, in the context with hepatocytes, that fasting followed by feeding reprograms metabolism in these cells by activating the IRE1-Xbp1s branch of UPR signaling. Xbp1s stimulates the UDP-galactose-4-epimerase (GalE) pathway, which promotes glucose assimilation rather than release from the hepatocytes (Deng et al., 2013). Such an outcome may be advantageous under pathological conditions like cancer, insulin resistance, and obesity because of reduced glucose availability in the blood. IRE1-Xbp1 mediated pathways are relevant to Longo's finding as both the components of the Longo's study—IGF1 and PKA—converge at some point of their signaling on the IRE1-Xbp1 axis of the UPR.

PKA is a biomarker for cancer and promotes cancer cell proliferation. It is known that PKA-mediated phosphorylation of its substrate causes lipolysis in cancer and obesity (Djouder et al., 2010), and one such substrate proposed recently is IRE1, where it is shown that phosphorylation of IRE1 by PKA (other than itself) up-regulates gluconeogenic genes and, thus, promotes hyperglycemia and glucose intolerance in obese mice (Mao et al., 2011). This evidence indirectly supports Longo's observation of lower PKA activity after fasting, which may arise from upstream signals and may retroactively regulate glucose levels. Interestingly, the insulin/IGF1 pathway regulates longevity in the cells. In *C. elegans*, the insulin/IGF1 pathway mutants have been shown to activate genes that promote longevity and ER stress resistance by IRE1-Xbp1 UPR signaling. Here, Xbp1 collaborates with FOXO-transcription factor DAF16 to bring the effect (Henis-Korenblit et al., 2010). This study addresses yet another side effect of chemotherapy—“senescence”—which the observed lower IGF1 levels in Longo's study may serve to counteract.

Moreover, two recent reports published around the same time highlight the role of IRE1-Xbp1 branch in SCs function. The first report shows that, with the induction of ER stress, the HSCs selectively undergo apoptosis while the closely related progenitors survive. The process occurs by the activation of distinct UPR branches in the two cell populations—PERK in the HSCs and IRE-Xbp1 in the progenitors, suggesting that the HSCs possess an intrinsic property to prevent propagation following damage brought through ER stress, and the IRE-Xbp1 branch serves to rescue the progenitors by an adaptive response (van Galen et al., 2014). Another rescuing effect of Xbp1 was shown in the second report. In the aging intestine of Drosophila melanogaster, the intestinal stem cells (ISCs) undergo enhanced proliferation and deficient differentiation resulting from chronic ER stress and reactive oxygen species (ROS) generation. The excessive ROS activates JNK, which in turn inhibits CncC (ortholog of mammalian Nrf2), an Xbp1 downstream transcription factor that (under normal conditions) would limit ROS accumulation and promote ISCs differentiation (Wang et al., 2014).

Our understanding of the role of fasting under metabolic diseases stands well elucidated, and, in cancer, where the mechanisms are more complex, the role of fasting in regulating SCs regeneration and self-renewal may seem quite intriguing, but it is nascent in the present state. So, it remains an open question as how to connect the means to an end. What are the signaling pathways up-regulated while fasting in SCs? What are the key players that can serve as a bridge between fasting and SC regeneration? Conceivably, UPR and its signaling branch, the IRE1-Xbp1 and its downstream signaling molecules play a significant role in SC regeneration. An upbeat approach would be to understand the modulation of these downstream targets such as NRF2, FOXO transcription factors, and the state of ROS generation while fasting in SCs (Figure 1). Rest assured, therapeutic measures designed around them instead would serve as better alternatives.

**Figure 1:**
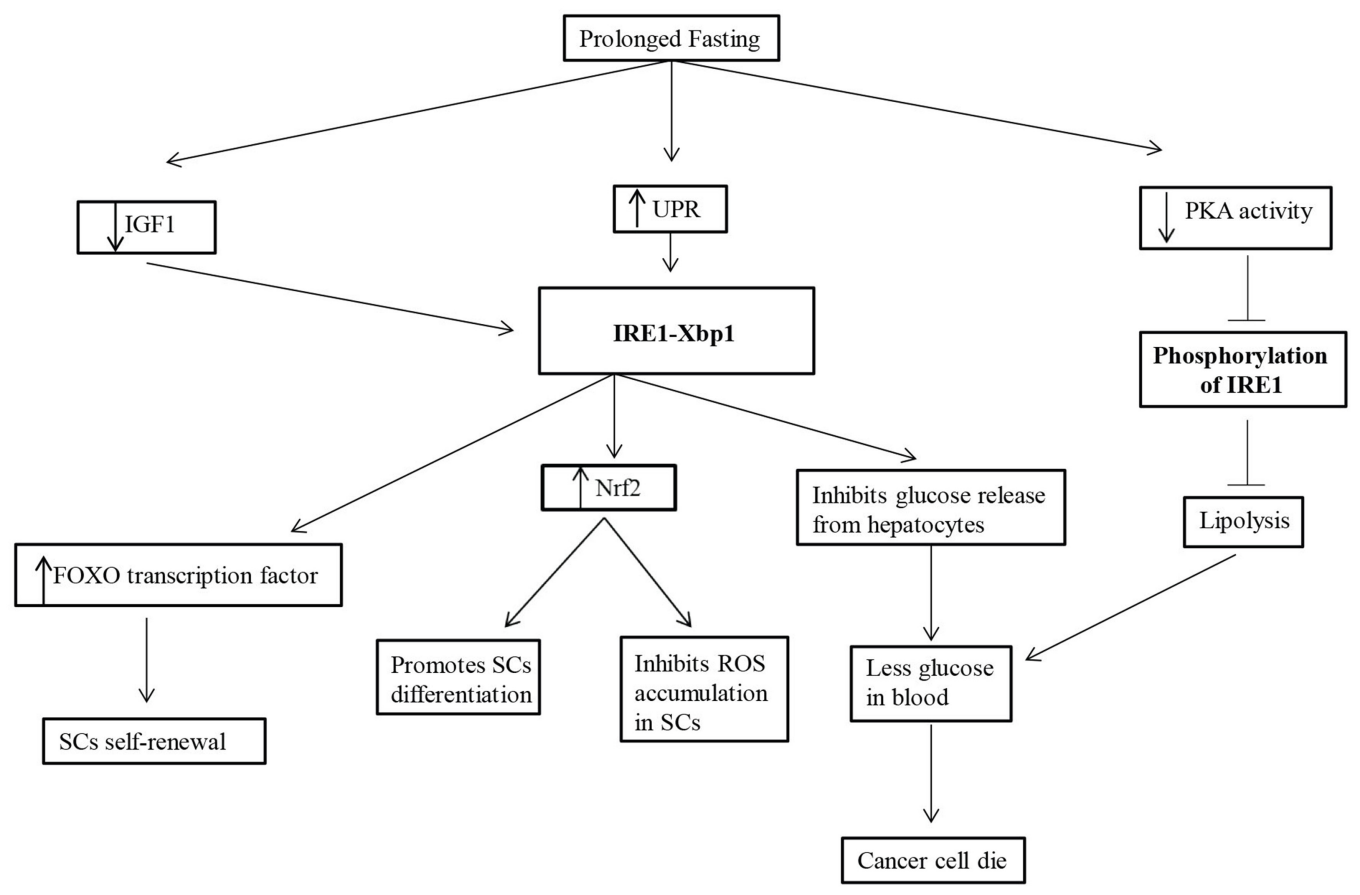
**Prolonged fasting upregulates the UPR, downregulates IGF1 signaling and decreases PKA activity**. The upregulated UPR activates IRE1-Xbp1 that inhibits glucose release from the hepatocytes leading to less glucose in the blood and thus cancer cell death, and activates Nrf2 that inhibits ROS accumulation and promotes differentiation of SCs. The decreased PKA activity leads to the inhibition of phosphorylation of IRE1 which in turn inhibits lipolysis and mediates in less glucose availability in the blood. The downregulated IGF1 signaling activates IRE1-Xbp1 that activates the FOXO transcription factors and stimulate SCs self-renewal.

## Conflict of interest statement

The author declares that the research was conducted in the absence of any commercial or financial relationships that could be construed as a potential conflict of interest.
